# Epidemiology of Chlamydia trachomatis, Mycoplasma genitalium, Neisseria gonorrhoeae, and Ureaplasma urealyticum in males of andrology clinics in Nanjing, China: a 10 years retrospective study

**DOI:** 10.3389/fcimb.2026.1852412

**Published:** 2026-06-24

**Authors:** Laiqing Zhu, Hang Lv, Xun Wang, Xue Zhou, Minhuan Li, Jun Kai, Liang Shi

**Affiliations:** 1 Department of Andrology, Nanjing Drum Tower Hospital, NanJing, Jiangsu, China; 2 Department of Laboratory Medicine, Nanjing Integrated Traditional Chinese and Western Medicine Hospital, NanJing, Jiangsu, China

**Keywords:** Chlamydia trachomatis (CT), Mycoplasma genitalium (MG), Neisseria gonorrhoeae (NG), Ureaplasma urealyticum (UU), the andrology outpatient clinic

## Abstract

**Objective:**

The aim of this single-center, cross-sectional and retrospective study is to assess the prevalence of Chlamydia trachomatis (CT), Mycoplasma genitalium (MG), Neisseria gonorrhoeae (NG), and Ureaplasma urealyticum (UU) among males of the Nanjing Drum Tower Hospital’s Andrology Department in Nanjing, China.

**Methods:**

In the review, we included male patients who consulted the Nanjing Drum Tower Hospital’s Andrology Department between 2016 and 2025. Urine samples were obtained from the patients, and all samples were tested by nucleic acid amplification tests (NAATs) for CT, MG, NG, and UU.

**Results:**

This research recruited 58,136 males (mean age: 32.29 ± 5.63 years; age range: 16–76 years). The positive rates for CT, MG, NG, and UU were 2.62%, 2.77%, 0.85%, and 50.16%, respectively. Males aged ≤ 24 years had the highest positive rates for CT (7.30%), MG (4.76%), and NG (4.89%), whereas males between the ages of 25–29 had the highest UU positive rate (53.17%). The single positive rate (93.84%) was significantly more common than mix positive rates (6.16%), alongside the MG+UU mix positive representing the most frequent combination. The ≤24-year age group had significantly more mix-positives than other age groups.

**Conclusions:**

Among males of our andrology outpatient clinic, the incidence of UU positive was markedly greater than that of CT, MG, and NG. Single positives were more predominant. Young people aged ≤24 had higher rates of STIs. Our findings contribute to a better understanding of the epidemiology of CT, MG, NG, and UU in our hospital of Nanjing, which allows for the comparison of patients across various geographies and sources, as well as the analysis of potential causes.

## Introduction

The transmission of sexually transmitted infections (STIs) constitutes a significant global health concern (Okunlola et al., 2025).The World Health Organization (WHO) reports that STIs impact millions of people every day (Gottlieb et al., 2024). It is widely recognized that certain males with sexually transmitted infections (STIs) may be asymptomatic, while others may exhibit symptoms such orchitis, epididymitis, or urethritis (Tuddenham et al., 2022). The symptomatic carriers of STIs among males of reproductive age can hinder detection and therapeutic intervention, heightening the likelihood of transfer to their partners. Infection with STIs can have detrimental impacts on semen quality, sperm production, fertilization, and pregnancy outcomes (Liu et al., 2021).

Chlamydia trachomatis (CT) infection is one of the most prevalent bacterial sexually transmitted diseases globally (Chiaradonna, 2008). It is a Gram-negative intracellular bacterium that contains DNA and RNA. It has a biphasic life cycle that includes an infectious extracellular form and a non-infectious, metabolically active intracellular form (Hocking et al., 2023).Clinical symptoms of CT infection in men may include epididymitis, orchitis, or reactive arthritis (Joly and Rayment, 2025).Mycoplasma genitalium (MG) is the smallest self-replicating prokaryote, and MG infections account for 15-20% of non-gonococcal urethritis (NGU) in males (Workowski et al., 2021). Tully initially identified and named MG in 1981 from the urethral secretions of males with NGU and conventional culture is challenging and time-consuming (Workowski et al., 2021; Jensen et al., 2022). MG infections are typically asymptomatic and non-pathogenic. Therefore, it is recommended that any patient suspected of having urethritis or pelvic inflammatory disease undergo a MG testing (Pinto-Sander and Soni, 2019). MG is transmitted through direct contact between genital mucous membranes, the penis and the anus, and oral-genital contact, highlighting the significance of multisite testing (Jensen et al., 2022; Kogler et al., 2025). Neisseria gonorrhoeae (NG) is a gram-negative diplococcus responsible for gonorrhoea, which ranks as the second most prevalent STI globally, following CT infection (Cumplido et al., 2025).The Ureaplasma urealyticum (UU) possesses a circular double-stranded DNA genome. It is the tiniest and most rudimentary self-replicating organism, positioned between bacteria and viruses, lacking a cell wall (Huang et al., 2024). UU mostly inhabits the human urogenital tract and is a prevalent bacterium associated with sexual activities in adults. Aside from a limited number of urogenital infections in men perhaps attributed to UU, there is less evidence indicating that UU is a significant contributor to urogenital infections in immunocompetent males (Stavart et al., 2023; Dai et al., 2026).Conventional diagnostic techniques exhibit low sensitivity, such as CT and MG are difficult to cultivate *in vitro*. Research indicates that NAATs demonstrate higher sensitivity and specificity in identifying genital tract pathogens through swabs from the vaginal tract or urine specimens (Son et al., 2025). Furthermore, it has been established that the detection rates of NG and CT in rectal swab samples utilizing NAATs are markedly superior to those of conventional diagnostic methods, including culture (Cosentino et al., 2012).

The incidence of various STIs, including CT, MG, NG, and UU, fluctuate significantly due to variations in customs, levels of education, lifestyles, and sexual health awareness among different nations and ethnic groups. In this study, our team conducted a single-center, cross-sectional and retrospective analysis to investigating the prevalence of four STIs across various geographic regions and age groups.

## Materials and methods

### Study population

A single-center, cross-sectional and retrospective analysis was performed on 58,136 patients who attended the Department of Andrology at Nanjing Drum Tower Hospital during the nearly 10-year period from 2016 to 2025. This study may include men getting normal health examinations, participating in voluntary screenings, or seeking fertility treatments. Patients may also be those exhibiting symptoms or possessing a clinical diagnosis. Only the result of first visit in our hospital will be taken into account for patients with multiple measurements. The Medical Ethics Committee at Nanjing Drum Tower Hospital has approved this study, which conforms to the Declaration of Helsinki and its latest revision(2024-988-001).

### Specimen collection

Obtain 2 mL of the first morning urine or the initial portion of urine following a minimum one-hour abstinence from urination, incorporate 2 mL of urine sample preservative, mix thoroughly, and utilize this as the testing sample. Before testing, researchers are necessary to keep the specimens in a refrigerator maintained at 2–8 degrees.

### RNA assay for four pathogens

Obtain urine samples from patients and analyze them with a nucleic acid detection kit (Shanghai Rendu Bio Co., Ltd.) to determine the existence of CT, MG, NG and UU pathogens in the samples. The detection of RNA from four pathogens consists of RNA extraction and isothermal amplification. In the RNA extraction process, RNA produced from bacterial lysis selectively adheres to magnetic particles in the nucleic acid extraction fluid. Pure target RNA is acquired by washing the magnetic particles, hence obviating the necessity for conventional centrifugation. The isolated RNA is identified via strand amplification technology (SAT), which integrates the functions of M-MLV reverse transcriptase, T7 RNA polymerase, and enhanced probe technology. Reverse transcriptase synthesizes a single DNA copy of the target RNA; subsequently, T7 RNA polymerase generates multiple RNA copies from this DNA; the optimized, fluorescently labelled probe selectively binds to these RNA copies, producing a fluorescent signal detected by the instrument. Testing results are analyzed according to the timing and strength of the real-time fluorescence signal, alongside positive and negative controls. All procedures comply rigorously to the manufacturer’s instructions included with the package. Every test performance comprises a positive control, a negative control, and an internal control. A positive result for pathogen RNA does not definitively signify an active infection, as the pathogen is frequently present in the urogenital tract of asymptomatic, healthy persons.

### Statistical analysis

SPSS Version 27.0 (SPSS Inc., Chicago, USA) was used for all analyses. Present the positive rates for pathogens as percentages. For contingency tables larger than 2×2, we used the Pearson chi-square test when ≤20% of expected cell counts were less than 5, and Fisher’s exact test when >20% of expected cell counts were less than 5. Associations were interpreted as: negligible (V < 0.10), weak (0.10 ≤ V < 0.30), moderate (0.30 ≤ V < 0.50), strong (0.50 ≤ V < 1.00).Apply the Bonferroni to adjust for *post hoc* pairwise comparisons across multiple groups. Accounting for multiple tests is important to reduce the risk of incorrect conclusions when testing across all comparisons, we performed 6 comparisons in the **Table 1**, 45 comparisons in the **Table 2**, 28 comparisons in the **Table 2** and 45 comparisons in the **Table 3**.These numbers are also shown below the tables. The significance level was adjusted by dividing 0.05 by the total number of comparisons. A p-value<0.05 is deemed statistically significant.

**Table 1:** The overall prevalence rates of CT,MG,NG and UU.

Pathogens	Prevalence(%)	χ2	p	Cramér’s V
CT	2.62% (1381/52842)	70046.39	<0.001	0.596
MG	2.77% (1587/57234)			
NG	0.85% (446/52520)^ab^			
UU	50.16% (17329/34547)^abc^			

a, p < 0.05 vs CT group. B, p < 0.05 vs MG group. c, p < 0.05 vs NG group.

Pearson's χ2 test; Pairwise comparisons between groups were performed using Pearson's χ2 test and adjusted for Bonferroni correction (m = 6).

**Table 2:** The prevalence of CT,MG,NG and UU infections in different years.

Years	CT	MG	NG	UU
2016	3.43% (215/6262)	4.47% (280/6264)	0.43% (27/6228)	60.14% (3766/6262)
2017	3.40% (234/6877)	3.71% (256/6876)	1.11% (76/6824)^a^	53.72% (2150/4002)^a^
2018	2.23% (159/7140)^ab^	2.33% (166/7134)^ab^	0.86% (61/7098)	53.19% (2462/4629)^a^
2019	2.92% (201/6886)	1.88% (129/6880)^ab^	0.60% (41/6836)	50.44% (2610/5174)^a^
2020	2.68% (149/5570)	2.17% (121/5571)^ab^	1.62% (89/5487)^acd^	47.77% (1994/4174)^abc^
2021	1.71% (31/1818)^ab^	2.55% (137/5368)^ab^	0.63% (28/4465)^e^	45.03% (2168/4815)^abcd^
2022	2.12% (85/4006)^ab^	2.97% (127/4269)^ad^	1.06% (28/2653)^a^	41.53% (907/2184)^abcde^
2023	2.02% (103/5090)^ab^	2.38% (130/5454)^ab^	0.68% (29/4265)^e^	43.13% (828/1920)^abcde^
2024	2.01% (93/4617)^ab^	2.02% (96/4741)^ab^	0.82% (35/4290)^e^	27.46% (123/448)^abcdefgh^
2025	2.43% (111/4576)	3.12% (146/4677)^ad^	0.66% (29/4374)^e^	34.19% (321/939)^abcdefgh^
χ2	63.97	139.79	70.14	638.56
p	<0.001	<0.001	<0.001	<0.001
Cramér’s V	0.035	0.049	0.037	0.136

a, p < 0.05 vs.2016 group. b, p < 0.05 vs. 2017 group. c, p < 0.05 vs 2018 group. d, p < 0.05 vs 2019 group. e, p < 0.05 vs 2020 group. f, p < 0.05 vs 2021 group. g, p < 0.05 vs 2022 group. h, p < 0.05 vs 2023 group. i, p < 0.05 vs 2024 group. Pearson’s χ2 test; Pairwise comparisons between groups were performed using Pearson’s χ2 test and adjusted for Bonferroni correction (m=45).

**Table 3:** The prevalence of CT,MG,NG and UU infections in different age groups.

Age (year)	CT	MG	NG	UU
≤24	7.30% (142/1944)	4.76% (97/2038)	4.89% (92/1882)	50.36% (708/1406)
25-29	2.80% (455/16258)^a^	2.81% (492/17505)^a^	0.89% (145/16252)^a^	53.17% (6062/11288)
30-34	2.33% (462/19818)^a^	2.39% (520/21741)^a^	0.46% (91/19805)^ab^	48.55% (6133/12669)^b^
35-39	2.25% (209/9301)^a^	2.91% (292/10054)^a^	0.71% (65/9206)^a^	48.06% (2712/5642)^b^
40-44	1.76% (63/3575)^ab^	2.89% (111/3835)^a^	0.68% (24/3509)^a^	50.50% (1133/2305)^b^
45-49	2.43% (32/1315)^a^	3.84% (54/1405)^c^	1.08% (14/1293)^ac^	49.88% (419/840)
50-59	2.86% (16/559)^a^	3.08% (18/584)	1.96% (10/511)^bcde^	43.23% (150/347)^b^
≥60	2.78% (2/72)	4.17% (3/72)	3.23% (2/62)	24.00% (12/50)^abcdef^
χ2	191.87	49.26	419.36	103.42
p	<0.001	<0.001	<0.001	<0.001
Cramér’s V	0.060	0.029	0.089	0.055

a, p < 0.05 vs.≤24 group. b, p < 0.05 vs. 25–29 group. c, p < 0.05 vs 30–34 group. d, p < 0.05 vs 35–39 group. e, p < 0.05 vs 40–44 group. f, p < 0.05 vs 45–49 group. g, p < 0.05 vs 50–59 group. Pearson's χ2 test; Pairwise comparisons between groups were performed using Pearson's χ2 test and adjusted for Bonferroni correction (m=28).

## Results

**Figure 1:**
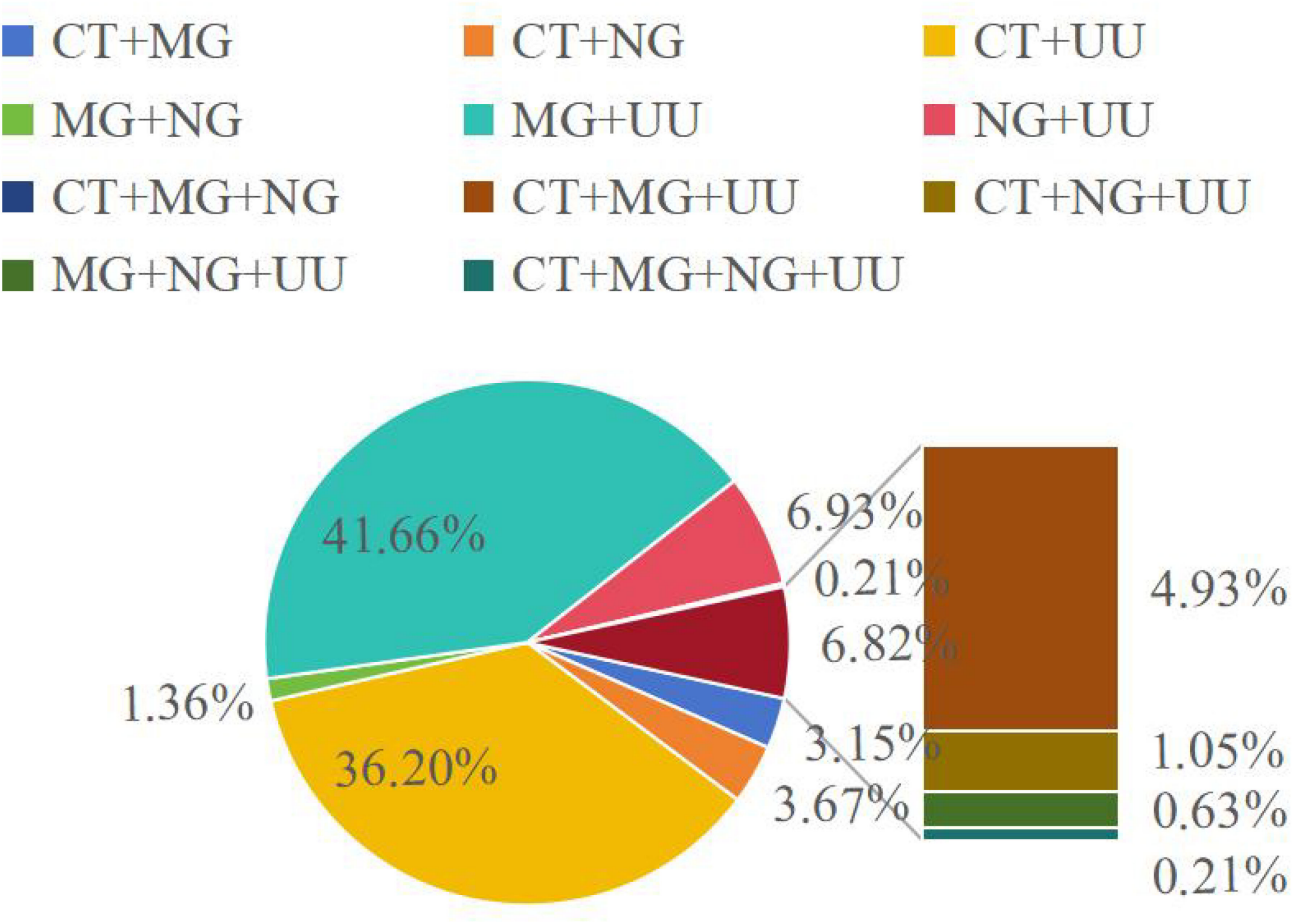
The Distribution of different mix-positive types.

In the retrospective review, we excluded males who underwent repeat testing after the initial round of testing and those with incomplete data; ultimately, we included 58,136 patients (mean age: 32.29 ± 5.63 years, range: 16–76 years). Patients were categorized according to the detected pathogens for overall analyzis(**Tables 3**-**4**). However, not all patients underwent testing for the four pathogens. There were 28,060 patients for whom all four pathogens were completely detected. This demographic was utilized for mix positive analysis (**Tables 5**–**7**; **Figure 1**). The denominator for the overall positive rate is the number of each pathogen testing over 10 years (**Table 1**). The positive rates of various pathogens in different years are determined using the denominator of all tested patients per pathogen per year (**Table 2**). The positive rates of various pathogens in different age groups are determined using the denominator of all tested patients per pathogen of each age groups (**Table 3**). The proportion of positive individuals in different years are determined using the denominator of patients for whom all four pathogens were completely detected per year (**Table 5**). The overall proportion of different mix positive types are determined using the denominator of total mix positive patients for whom all four pathogens were completely detected over 10 years (**Figure 1**). The proportion of single positive and different mix positive types in different years or ages are determined using the denominator of the total positive patients for whom all four pathogens were completely detected per year or ages groups (**Tables 6**, **7**). Demographic information on the patients enrolled annually is reported in **Table 4**.

**Table 4:** The demographic information of the enrolled patients.

Years	N	Age (year)
2016	6268	32.51 ± 6.12
2017	6884	31.86 ± 6.00
2018	7142	31.78 ± 5.67
2019	6886	31.69 ± 5.45
2020	5575	31.75 ± 5.40
2021	5441	32.08 ± 5.36
2022	4401	32.39 ± 5.55
2023	5531	32.86 ± 5.46
2024	5011	33.23 ± 5.46
2025	4997	33.32 ± 5.40
Total	58136	32.29 ± 5.63

**Table 5:** The proportion of infection individuals in different years.

Years	single positive(n)	mix positive(n)	total positive(n)	total(n)	Single positive/total positive (%)	mix positive /total positive (%)	positive individuals (%)	χ2	p	Cramér’s V
2016	3617	310	3927	6212	92.11%	7.89%	63.22%	410.04	<0.001	0.121
2017	2125	171	2296	3927	92.55%	7.45%	58.47%^a^			
2018	2446	126	2572	4575	95.10%	4.90%	56.22%^a^			
2019	2598	139	2737	5118	94.92%	5.08%	53.48%^ab^			
2020	1998	99	2097	4077	95.28%	4.72%	51.43%^abc^			
2021	415	24	439	939	94.53%	5.47%	46.75%^abcd^			
2022	493	28	521	1168	94.63%	5.37%	44.61%^abcde^			
2023	578	26	604	1346	95.70%	4.30%	44.87%^abcde^			
2024	46	6	52	157	88.46%	11.54%	33.12%^abcde^			
2025	210	24	234	541	89.74%	10.26%	43.25%^abcde^			
Total	14526	953	15479	28060	93.84%	6.16%	55.16%			

a, p < 0.05 vs. 2016 group. b, p < 0.05 vs. 2017 group. c, p < 0.05 vs 2018 group. d, p < 0.05 vs 2019 group. e, p < 0.05 vs 2020 group. f, p < 0.05 vs 2021 group. g, p < 0.05 vs 2022 group. h, p < 0.05 vs 2023 group. i, p < 0.05 vs 2024 group. Pearson’s χ2 test; Pairwise comparisons between groups were performed using Pearson’s χ2 test and adjusted for Bonferroni correction (m=45).

**Table 6:** The single positive and the different mix positive types in different years.

Years	single positive	CT+MG	CT+NG	CT+UU	MG+NG	MG+UU	NG+UU	CT+MG+NG	CT+MG+UU	CT+NG+UU	MG+NG+UU	CT+MG+NG+UU
2016	92.11% (3617/3927)	0.25% (10/3927)	0.00% (0/3927)	2.83% (111/3927)	0.00% (0/3927)	4.20% (165/3927)	0.23% (9/3927)	0.00% (0/3927)	0.33% (13/3927)	0.05% (2/3927)	0.00% (0/3927)	0.00% (0/3927)
2017	92.55% (2125/2296)	0.30% (7/2296)	0.52% (12/2296)	1.92% (44/2296)	0.13% (3/2296)	3.18% (73/2296)	0.44% (10/2296)	0.04% (1/2296)	0.57% (13/2296)	0.17% (4/2296)	0.09% (2/2296)	0.09% (2/2296)
2018	95.10% (2446/2572)	0.04% (1/2572)	0.19% (5/2572)	1.98% (51/2572)	0.12% (3/2572)	1.83% (47/2572)	0.58% (15/2572)	0.04% (1/2572)	0.08% (2/2572)	0.04% (1/2572)	0.00% (0/2572)	0.00% (0/2572)
2019	94.92% (2598/2737)	0.26% (7/2737)	0.15% (4/2737)	2.19% (60/2737)	0.04% (1/2737)	1.79% (49/2737)	0.37% (10/2737)	0.00% (0/2737)	0.26% (7/2737)	0.04% (1/2737)	0.00% (0/2737)	0.00% (0/2737)
2020	95.28% (1998/2097)	0.10% (2/2097)	0.10% (2/2097)	1.81% (38/2097)	0.14% (3/2097)	1.34% (28/2097)	0.72% (15/2097)	0.00% (0/2097)	0.33% (7/2097)	0.05% (1/2097)	0.14% (3/2097)	0.00% (0/2097)
2021	94.53% (415/439)	0.23% (1/439)	0.23% (1/439)	1.82% (8/439)	0.46% (2/439)	1.59% (7/439)	0.46% (2/439)	0.00% (0/439)	0.46% (2/439)	0.23% (1/439)	0.00% (0/439)	0.00% (0/439)
2022	94.63% (493/521)	0.00% (0/521)	0.38% (2/521)	2.30% (12/521)	0.00% (0/521)	1.92% (10/521)	0.38% (2/521)	0.00% (0/521)	0.19% (1/521)	0.00% (0/521)	0.19% (1/521)	0.00% (0/521)
2023	95.70% (578/604)	0.00% (0/604)	0.00% (0/604)	1.82% (11/604)	0.17% (1/604)	1.99% (12/604)	0.17% (1/604)	0.00% (0/604)	0.17% (1/604)	0.00% (0/604)	0.00% (0/604)	0.00% (0/604)
2024	88.46% (46/52)	0.00% (0/52)	5.77% (3/52)	3.85% (2/52)	0.00% (0/52)	0.00% (0/52)	1.92% (1/52)	0.00% (0/52)	0.00% (0/52)	0.00% (0/52)	0.00% (0/52)	0.00% (0/52)
2025	89.74% (210/234)	0.85% (2/234)	2.56% (6/234)	3.42% (8/234)	0.00% (0/234)	2.56% (6/234)	0.43% (1/234)	0.00% (0/234)	0.43% (1/234)	0.00% (0/234)	0.00% (0/234)	0.00% (0/234)

**Table 7:** The single positive and the different mix positive types in different ages.

Age	single positive	CT+MG	CT+NG	CT+UU	MG+NG	MG+UU	NG+UU	CT+MG+NG	CT+MG+UU	CT+NG+UU	MG+NG+UU	CT+MG+NG+UU
≤24	87.31% (626/717)	0.28% (2/717)	1.53% (11/717)	5.30% (38/717)	0.70% (5/717)	3.07% (22/717)	1.12% (8/717)	0.00% (0/717)	0.28% (2/717)	0.28% (2/717)	0.14% (1/717)	0.00% (0/717)
25-29	94.06% (5197/5525)	0.29% (16/5525)	0.16% (9/5525)a	2.19% (121/5525)	0.07% (4/5525)	2.32% (128/5525)	0.47% (26/5525)	0.02% (1/5525)	0.31% (17/5525)	0.09% (5/5525)	0.00% (0/5525)	0.02% (1/5525)
30-34	94.57% (4929/5212)	0.10% (5/5212)	0.13% (7/5212)a	2.13% (111/5212)	0.04% (2/5212)	2.34% (122/5212)	0.31% (16/5212)	0.00% (0/5212)	0.33% (17/5212)	0.00% (0/5212)	0.06% (3/5212)	0.00% (0/5212)
35-39	93.53% (2298/2457)	0.16% (4/2457)	0.24% (6/2457)	1.99% (49/2457)	0.04% (1/2457)	3.34% (82/2457)	0.37% (9/2457)	0.00% (0/2457)	0.20% (5/2457)	0.12% (3/2457)	0.00% (0/2457)	0.00% (0/2457)
40-44	95.01% (972/1023)	0.10% (1/1023)	0.00% (0/1023)	1.56% (16/1023)	0.00% (0/1023)	2.44% (25/1023)	0.29% (3/1023)	0.00% (0/1023)	0.39% (4/1023)	0.00% (0/1023)	0.10% (1/1023)	0.10% (1/1023)
45-49	92.78% (360/388)	0.52% (2/388)	0.52% (2/388)	2.06% (8/388)	0.00% (0/388)	3.35% (13/388)	0.52% (2/388)	0.00% (0/388)	0.26% (1/388)	0.00% (0/388)	0.00% (0/388)	0.00% (0/388)
50-59	91.67% (132/144)	0.00% (0/144)	0.00% (0/144)	1.27% (2/144)	0.64% (1/144)c	3.18% (5/144)	0.69% (1/144)	0.64% (1/144)	0.64% (1/144)	0.00% (0/144)	0.64% (1/144)	0.00% (0/144)
≥60	92.31% (12/13)	0.00% (0/13)	0.00% (0/13)	0.00% (0/13)	0.00% (0/13)	0.00% (0/13)	7.69% (1/13)	0.00% (0/13)	0.00% (0/13)	0.00% (0/13)	0.00% (0/13)	0.00% (0/13)

### The overall prevalence rates of four STIs in males

The total amount of CT, MG, NG, and UU tests conducted from 2016 to 2025 was 52,842, 57,234, 52,520, and 34,547, respectively. UU exhibited the greatest prevalence rates among males at 50.16%, followed by MG at 2.77%, CT at 2.62%, and NG with the lowest rate at 0.85%. A comparison of the prevalence rates of the four pathogens revealed highly significant statistical differences across the groups (χ² = 70,046.39, *p* < 0.001) applying chi-square test. The Cramér’s V suggested a strong association, V = 0.596.The *post hoc* pairwise comparisons indicated that the prevalence rate in the UU group was significantly elevated in contrast to the other three groups(all *p* < 0.05); the prevalence rate in the NG group decreased significantly in relation to the CT and MG groups (all *p* < 0.05); and no statistically significant variation in prevalence rates was seen among the CT and MG groups.(*p* > 0.05)(**Table 1**).

### The prevalence of CT,MG,NG and UU positives in different years

The annual prevalence rates varied for CT between 1.71% and 3.43%, for MG between 1.88% and 4.47%, for NG between 0.43% and 1.62%, and for UU between 27.46% and 60.14%. The differences in CT prevalence rates across the years were statistically significant (χ² = 63.97, *p* < 0.001). The Cramér’s V suggested a negligible association, V = 0.035. The *post hoc* pairwise comparisons denoted that the prevalence rates in 2016 and 2017 were significantly higher compared to most other years, with the exception of 2019,2020 and 2025 (p < 0.05). The MG prevalence rate exhibited statistically significant differences over the years (χ² = 139.79, *p* < 0.001). The Cramér’s V suggested a negligible association, V = 0.049. The prevalence initially declined from 4.47% in 2016, subsequently experiencing a slight rebound to 3.12% in 2025, demonstrating overall fluctuating changes; The prevalence rates from 2018 to 2024 were significantly lower than those in 2016 and 2017 (*p* < 0.05), according to *post hoc* pairwise comparisons; the prevalence rate of 2025 was significantly lower than that of 2016 (*p* < 0.05) but did not differ significantly compared to 2017. Despite the NG prevalence rate being consistently at a low level, the annual variations were statistically significant (χ² = 70.14, *p* < 0.001); The Cramér’s V suggested a negligible association, V = 0.037. The *post hoc* pairwise comparisons denoted that the prevalence rates in 2020 was significantly higher than in most other years, except for 2017 and 2022 (*p* < 0.05); The prevalence rate in 2017 was considerably greater than that of 2016(*p* < 0.05), with no significant changes noted in comparison to other years. The UU positive rate exhibited statistically significant variations over the years (χ² = 638.56, *p* < 0.001), demonstrating a consistent and prominent decline; The Cramér’s V suggested a weak association, V = 0.136. The *post hoc* pairwise comparisons indicated that the prevalence rates for each year from 2017 to 2025 were significantly lower than that of 2016 (*p* < 0.05), and the prevalence rates further decreased from 2020 to 2025 (*p* < 0.05) (**Table 2**).

### The prevalence of CT,MG,NG and UU in different age groups

The positive rate of CT shown a statistically notably variability among age groups (χ² = 191.87, *p* < 0.001), with the overall rate declining as age increased. The Cramér’s V suggested a negligible association, V = 0.060. The *post hoc* pairwise comparisons revealed that the positive rate was highest in the ≤24 years group (7.30%), and the infection rates in all other age groups were significantly lower than that of the ≤24 years group (all *p* < 0.05). A significant difference was noted between the 40–44 age group and the 25–29 age group (p < 0.05); but there were no noticeable differences between the other groups. The differences in MG prevalence rates among various age groups were statistically significant (χ² = 48.99, p < 0.001). The Cramér’s V suggested a negligible association, V = 0.029. The ≤24 age group had the greatest prevalence rate (4.76%), which was substantially higher than the 25-29, 30-34, 35-39, and 40–44 age groups (all *p* < 0.05). The 30–34 and 45–49 age groups had significantly different MG prevalence rates (p < 0.05), whereas the other age groups did not differ significantly. The differences in NG prevalence rates among various age groups were statistically significant (χ² = 418.29, *p* < 0.001). The Cramér’s V suggested a negligible association, V = 0.089. The ≤24 age group had the highest prevalence rate (4.89%), followed by the ≥60 age group (3.23%). The age categories of 25–29 and 30–34 showed a significant difference (*p* < 0.05), while the other groups showed no significant differences. Despite the UU prevalence rate remained at a relatively high level among all age, the differences between these groups were statistically significant (χ² = 96.96, *p* < 0.001). The Cramér’s V suggested a negligible association, V = 0.055. The 25–29 age group had the greatest UU prevalence rate (53.17%), which differed significantly from the 30-34, 35-39, 50–59 and ≥ 60 age groups (all p < 0.05).The ≥60 age group differed significantly with the other group (**Table 3**).

### The positive types in different years

Of the 28,060 males assessed for all four pathogens, 55.16% of males were detected with at least one pathogen. There was a notable variation in the proportion of positive individuals(total positive/total individuals) across different years (χ² = 410.04, p < 0.001). The Cramér’s V suggested a weak association, V = 0.121. From 2016 to 2021, the overall trend showed a fall, dropping from 63.22% in 2016 to 46.75% in 2021, followed by stabilization from 2022 to 2025. The proportion of patients with single positives caused by CT, MG, NG, or UU was 93.84% (range: 88.46%-95.70%), whereas the proportion of patients with mix positives was less than 10% (**Table 5**).

### The distribution of different mix positive types

Among patients with mix positives, the predominant combination was concurrent MG+UU positive (41.66%), followed by simultaneous CT+UU positive (36.20%). The rarest combinations were CT+MG+NG positive (0.21%) and CT+MG+NG+UU positive (0.21%) (**Figure 1**).

### The single positive and the different mix positive types in different years and different ages

The proportion of single positives has consistently surpassed that of mixed positives, regardless of the years or ages. The most frequent mix positive type was CT+UU and MG+UU. The proportion of CT +NG were elevated in 2024 and 2025 compared to previous years, with the highest prevalence rate for CT+NG recorded in 2024 at 5.77%. The prevalence of MG + UU was the lowest in 2024. The prevalence of single positives in the ≤24 age group was lower than other age groups. The prevalence of CT+NG, CT+UU and MG + NG mix positives in the ≤24 age group was elevated compared to other age groups. The proportion of triple or four positives with different pathogens was lower than singer positive or two positives (**Tables 6**, **7**).

## Discussion

While there have been numerous epidemiological studies on STIs in women, such research is rather limited for men. This retrospective study focused on the prevalence of CT, MG, NG, and UU in males at an andrology outpatient clinic during an extensive time period. We specifically analyzed the prevalence of infections attributable to these four pathogens in a cohort of 58,136 patients treated at Nanjing Drum Tower Hospital in Nanjing, China, spanning a nearly decade-long period from 2016 to 2025.Furthermore, we analyzed the proportion of these pathogens of various age groups and the rate of mix positive among them.

CT is the primary etiological agent of bacterial STIs worldwide (Joly and Rayment, 2025). According to a latest meta-analysis, 2.9% (95% CI: 2.4%-3.5%) of the general population worldwide has a CT infection (Bragazzi et al., 2025).A further meta-analysis reported a CT infection rate of 20.6% among men with infertility and demonstrated that CT infection substantially elevates the chance of male infertility (Keikha et al., 2022).This survey indicates that the CT infection rate is 2.62%, which is at the lower spectrum of global reports (Bragazzi et al., 2025).Compared with domestic studies, this result is close to the 3.11% identified in secretion samples at the Putian Reproductive Center in Fujian (Zeng et al., 2024), and exceeds the reported rates among men of reproductive age in Chengdu (1.47%) and Chongqing (1.93%) (Li et al., 2025; Luo et al., 2025), yet significantly lower than the rates observed in outpatients surveys of men in Kunming (11.70%), Shanghai (8.36%), and Jingzhou (13.54%) based on urogenital swabs (Wang et al., 2023; Liu et al., 2024; Wei et al., 2025).Compared with global data, the prevalence rate in this study was higher than that observed in screening of the general population in many European countries (1.75% in Belgium, 1.0% in Germany, and 0.5% in Slovenia), but significantly lower than that in certain high-risk subpopulations in Africa and South America (9.3% among students in Tanzania and 14.5% among homeless people in Colombia) (Fischer et al., 2021; Gassowski et al., 2022; Klavs et al., 2022; Mcharo et al., 2022; Vélez-Gómez et al., 2022).It may be inferred that the CT infection rate is influenced by geographical location, population characteristics, and the type of samples tested. This study revealed that the CT incidence was highest among those aged ≤24, aligning with findings from studies in Slovenia and several Chinese cities, such as Jingzhou (Klavs et al., 2022; Liu et al., 2024; Li et al., 2025; Luo et al., 2025; Wei et al., 2025).A German study reveals that the infection prevalence is elevated among individuals aged 25 to 29 (Gassowski et al., 2022).Cluster analysis by experts has verified that, in both Europe and China, the infection rate of CT among individuals aged 24 and under exceeds that of older age groups (Cai et al., 2022; Joly and Rayment, 2025).

Mycoplasma genitalium (MG) is a pathogen responsible for STIs, and there is limited epidemiological data on MG (Yusuf et al., 2023). According to a meta-analysis,the infection rate of MG was 1.3% in a random sample of the general population in high-income nations and 3.9% in low-income countries, with no significant gender difference (Baumann et al., 2018). In this survey, the MG prevalence rate was 2.7%, which is higher than that reported in a population-based survey in Slovenia (0.5%), but lower than rates observed in STI outpatient clinics in Guangdong (7.4%) and in male outpatients in Hangzhou (5.26%) and Chongqing (3.57%) (Zhang et al., 2021; Luo et al., 2025; Qiu et al., 2026). Our study revealed the highest prevalence of MG in the cohort aged ≤24 years, which is similar to findings from a study in Chongqing (Luo et al., 2025), but contrasting with those in Hangzhou (ages 21-30) and Slovenia (ages 25-34) (Klavs et al., 2022; Qiu et al., 2026).MG is a prevalent STI pathogen among high-risk populations; however, testing only urine samples for MG in these populations reduces the detection rate (Jensen et al., 2022; Tran et al., 2026).A MG screening study conducted by four collaborative from four countries found that the MG infection rate in rectal specimens (12.5%) surpassed that in urine specimens of males (3.9%; p<0.001). The study additionally advised that MSM(men who have sex with men) consider rectal sampling (Perry et al., 2022).A study conducted at a STI clinic in Gdańsk, Poland, found that urine samples were the predominant source of MG testing for non-MSM patients, whereas rectal samples were the primary source for MSM patients (Kadylak et al., 2024).

A study indicates that the prevalence of NG among infertile individuals worldwide is approximately1.5% in males, in contrast to roughly 0.8% in the overall population (Chemaitelly et al., 2021). The prevalence rate of NG in this study was 0.85%, markedly less than the rates documented in patient studies from Kunming (Wang et al., 2023; Liu et al., 2024; Tian et al., 2024; Wei et al., 2025), but higher than that reported in male outpatient or reproductive health surveys in Putian (0.33%), Chengdu (0.11%), and Chongqing (0.12%) (Zeng et al., 2024; Li et al., 2025; Luo et al., 2025).Compared with international studies, the NG positive rate in this study was lower than that among students in Tanzania (1.5%) and homeless populations in Colombia (21.3%) (Mcharo et al., 2022; Vélez-Gómez et al., 2022),but significantly greater than that of Slovenia’s overall population (0%) (Klavs et al., 2022).Our study found the NG positive rate was greatest in the group of individuals aged ≤24 years (4.89%), followed by the ≥60years group (3.23%). This discovery is in accordance with the findings of a survey of males in Jingzhou (Liu et al., 2024), whereas investigations conducted in Hangzhou, Chongqing and Chengdu have shown no significant differences in NG infection rates across different age groups (Tian et al., 2024; Li et al., 2025; Luo et al., 2025).

The literature lacks definitive evidence linking UU to NGU and testing can be considered in the absence of alternative infections (Ligero-López et al., 2025).In this study, the prevalence of UU was 50.16%, which is higher than that reported in surveys of urology outpatient clinics or relevant populations in many regions across China, such as Kunming (33.31%), Shanghai (33.36%), Putian (35.68%), Hangzhou (28.54%), Jingzhou (24.50%), and Chengdu (41.00%) (Wang et al., 2023; Liu et al., 2024; Tian et al., 2024; Zeng et al., 2024; Li et al., 2025; Wei et al., 2025). This study identified the greatest positive proportion of UU was exhibited in the 25–29 age cohort., which differs from the findings conducted in Kunming (30–34 years) and Chengdu (36–45 years). However, the prevalence in the≥60 age group (24.00%) was lower than in other age groups, which is in accordance with the finds of the researches in those two cities (Li et al., 2025; Wei et al., 2025).

The study found the positive rates for CT and MG in 2016 and 2017 were significantly higher than in other years. Although UU showed a general decline trend, NG was significantly higher in 2017 and 2020 compared to other years. In Jingzhou, China, the prevalence of CT infection from 2018 to 2022 was significantly higher in 2018 than in 2020- 2022. No notable difference was observed in the prevalence rate of NG positive across the different years, while the prevalence of UU was higher in 2018 and 2019 than in 2020-2022 (Liu et al., 2024).In Kunming, China, the prevalence for CT and NG exhibited relative stability throughout the study period(2018-2024), whereas UU showed a significant increase in 2022 and maintained stable in other years (Wei et al., 2025).A study conducted in Chongqing, China, revealed that the annual prevalence for CT and NG remained stable throughout the study period(2018-2022); the prevalence rate for MG was decreased in both 2020 and 2022 compared to 2018, 2019, and 2021 (Luo et al., 2025). The frequency of infection with MG in Hangzhou, China, remained stable between 2020 and 2023, consistent with our study. Our research showed a general decline trend in UU testing, especially during 2024 and 2025 (**Table 2**). Upon investigation, we found that doctors had acknowledged UU as a colonizing bacterium with no definitive link to genital tract infections; thus, they ceased recommending testing or research on it, consistent with guidelines from Europe, the United States, and other areas (Stavart et al., 2023).

Our study revealed significant variation in the overall positive rates of the four distinct pathogens, alongside a strong association among them(V = 0.596). This finding has important implications for reflecting the local pathogen spectrum and population susceptibility patterns. In different years and age groups, the p-value implied a statistically significant difference; however, the negligible or week associations implied that year or age is a meaningful but not dominant influence element influencing variations in positive rates. The primary reason for the detected significant difference may be the large sample size. Additional research combining health examination surveys, molecular epidemiology, and behavioral risk assessments is necessary to clarify the underlying factors influencing these trends.

In this study, the population included in the calculation of annual positive rates for different pathogens consisted of all patients. However, when calculating the proportion of positive individuals across different years, the population included only those tested for all four pathogens, as the proportion of positive individuals would be biased if calculated utilizing patients who were tested for fewer than four pathogens. Our study found that the proportion of positive individuals showed an overall downward trend between 2016 and 2021, stabilizing between 2022 and 2025. The finding may be directly correlated with the persistent decrease in population of those tested for all four pathogens and infection rates in UU. Since UU is predominant among positive individuals, the drop in testing volume has led to a decrease in the proportion of positive individuals.

This study used a population in which all four pathogens were tested to determine the proportion of individuals with single positives and mix positives, thereby ensuring the data validity. This study found that positives cases were primarily caused by a single pathogen, while mix positives being less infrequent. Our research showed that the persistent decrease in the quantity of UU tests has resulted in a progressively diminishing population of patients evaluated for all four pathogens. Regarding the number of individuals tested by UU and its intrinsically elevated positive rate, the proportion of positive patients remained relatively decrease (**Tables 5**, **6**). In this study, mix positives accounted for 6.16% of all infections; the most common combination was MG+UU, succeeded by CT+UU and NG+UU. All studies on the detection of CT, NG, and UU pathogens in Chengdu, Shanghai, and Kunming,discovered that the most prevalent combination was CT+UU mix positives, consistent with the distribution features of CT,NG and UU observed in the study (Wang et al., 2023; Li et al., 2025; Wei et al., 2025). A study conducted in Chongqing discovered the predominant combination was CT+MG mix positives by detecting CT, MG, and NG in patients. This finding contradicts our study, in which the most common combination among the three tests (CT, MG, and NG) was CT+NG (Luo et al., 2025). Mixed infections primarily consist of mix positives involving UU and other pathogens. Research have shown that UU readily colonizes the urethra but does not necessarily cause urethritis. Moreover, UU may function as an opportunistic pathogen, reproducing rapidly when other pathogens cause inflammation and the urethral dysbacteriosis (Jordan et al., 2020). The CT+NG mix positive observed in this study is also an important combination. A study have suggested that mucosal damage caused by NG may enhance CT adhesion and immune evasion, particularly among sexually active young adults (Darville, 2021; Zhai et al., 2022). However, the exact mechanism remains unclear, so further research may shed new light on our currently limited understanding of STIs. This study revealed that the rate of CT + NG mix positive was substantially increased in 2024 and 2025 than in other years. In our study, the proportion of mix positive was markedly greater in the ≤24 age group compared to other age groups, consistent with findings from a study in Zhuhai, China (Zhang et al., 2021),and partially consistent with finding from Hangzhou,where the significant prevalence of mix positive was noted in the ≤20 age group (Tian et al., 2024). A review of several studies suggests that the reasons why young people are more susceptible to infection may include an increased number of sexual partners, insufficient knowledge regarding sexual health, and a lack of specific immunity (Fischer et al., 2021; Bozicevic et al., 2023; Tian et al., 2024). The study population for analyzing mix positive rates comprised individuals who tested positive for all four pathogens. The specific tests conducted may not align completely with those in other studies, thus introducing bias in the interpretation of our results. In addition, only a portion of our study’s participants underwent testing for all four pathogens, which could create bias when estimating the number of infections and the proportion of mixed infections. Nonetheless, this still generally indicates certain patterns of co-occurrence.

The prevalence of STIs exhibits considerable diversity, attributable to numerous factors. Firstly, the prevalence of STIs varies greatly among different populations, such as the general population, transgender and gender-diverse individuals(TG), men sex workers (MSWs), and men who have sex with men (MSM) (Tuddenham et al., 2022). The absence of participation from high-risk groups in general population screening may lead to a drop prevalence of these infections (Klavs et al., 2022). Our study population comprises patients from an andrology clinic, partly men pursuing fertility treatment; hence, the positive rate may be inferior to that observed at sexually transmitted disease clinic.Secondly, the principal sample types used for detecting sexually transmitted pathogens encompass urine, rectal swabs, genital swabs, and throat swabs for males. The detection rates may vary depending on the sampling method. The type of sample utilized in our study was urine. Many studies recommend that routine combined testing of genital, rectal, and oropharyngeal specimens to improve detection rates, to prevent missed diagnoses, and reduce workload, time, and financial costs (Lily Aboud et al., 2021; Kogler et al., 2025; Tran et al., 2026).

## Limitations

This study was conducted exclusively in the Department of Andrology at Nanjing Drum Tower Hospital. The lack of multicenter data from Nanjing precludes a comprehensive assessment of the epidemiology of these four pathogens in the Nanjing area of China. The participants in this study were from the hospital’s andrology department and included those undergoing routine health examinations, voluntary screening, or men seeking fertility services, or they may have been patients with symptoms or clinical diagnoses. Due to insufficient outpatient medical records and the absence of sexual questionnaires, we could not assess the occurrence of genital infections among patients with varying clinical diagnoses and diverse sexual behaviors. The study utilized urine specimens and did not collect samples from multiple sites, which may have resulted in underestimation of infection rates. The lack of an epidemiological questionnaire regarding sexual behavior and detailed outpatient medical records prevented us from thoroughly exploring potential risk factors. Subsequent studies will further address these issues.

## Conclusion

This study presents epidemiological information regarding CT, MG, NG, and UU infections among males at an outpatient urology clinic in Nanjing, China. The results indicate that UU is the most commonly detected organism, succeeded by CT and MG, with NG being the least prevalent. Single infections were predominant. Young men ≤ 24 years exhibited the highest prevalence of STIs. In the future, we will be imperative to conduct large-scale research that encompasses behavioral data that are associated with STIs, as well as multiple centers and sampling sites.

## Data Availability

The original contributions presented in the study are included in the article/supplementary material. Further inquiries can be directed to the corresponding author.
